# Supplementary material for: Effects of Deformed Wing Virus-Targeting dsRNA on Viral Loads in Bees Parasitised and Non-Parasitised by Varroa destructor
Source: Viruses. 2023 Nov 15;15(11):2259. doi: 10.3390/v15112259 (PMC10674661; doi:10.3390/v15112259)
Supplement: Supplementary file 1 [file viruses-15-02259-s001.zip › FigureS1.pdf]

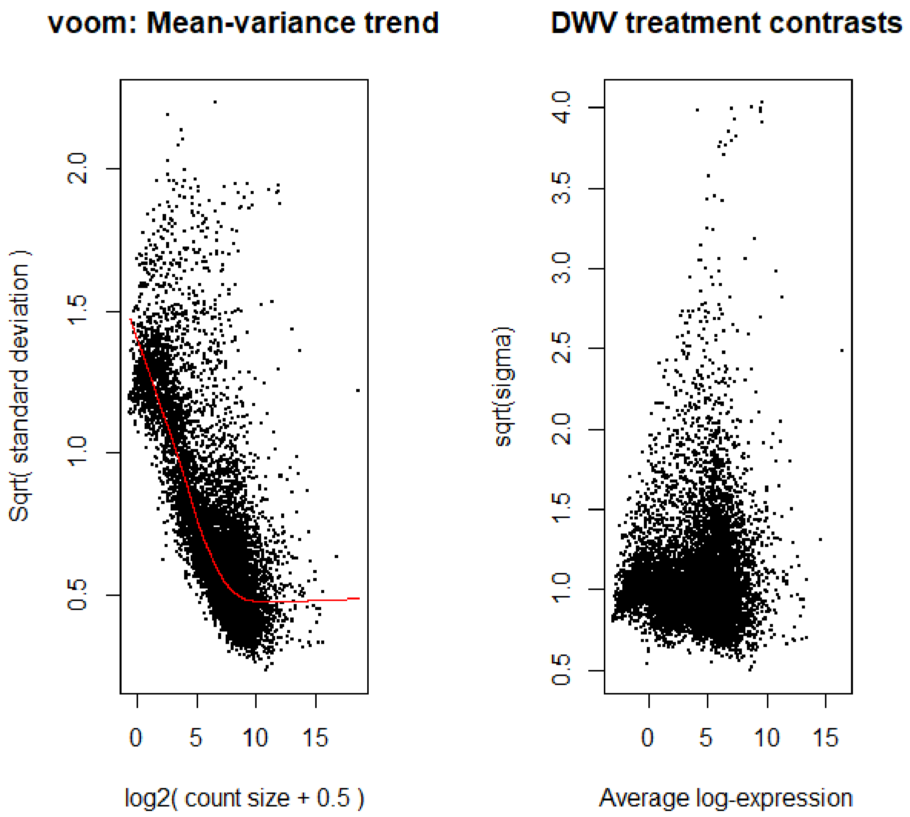

Figure S1. Voom output showing the mean-variance relationships for each gene (left) and linear modelling of the mean-variance relationships with contrasts investigated between control-dsRNA vs DWV-dsRNA, sugar control vs DWV-dsRNA, and sugar control vs control-dsRNA (right).
