# Supplementary material for: Effects of Deformed Wing Virus-Targeting dsRNA on Viral Loads in Bees Parasitised and Non-Parasitised by Varroa destructor
Source: Viruses. 2023 Nov 15;15(11):2259. doi: 10.3390/v15112259 (PMC10674661; doi:10.3390/v15112259)
Supplement: Supplementary file 1 [file viruses-15-02259-s001.zip › FigureS2.pdf]

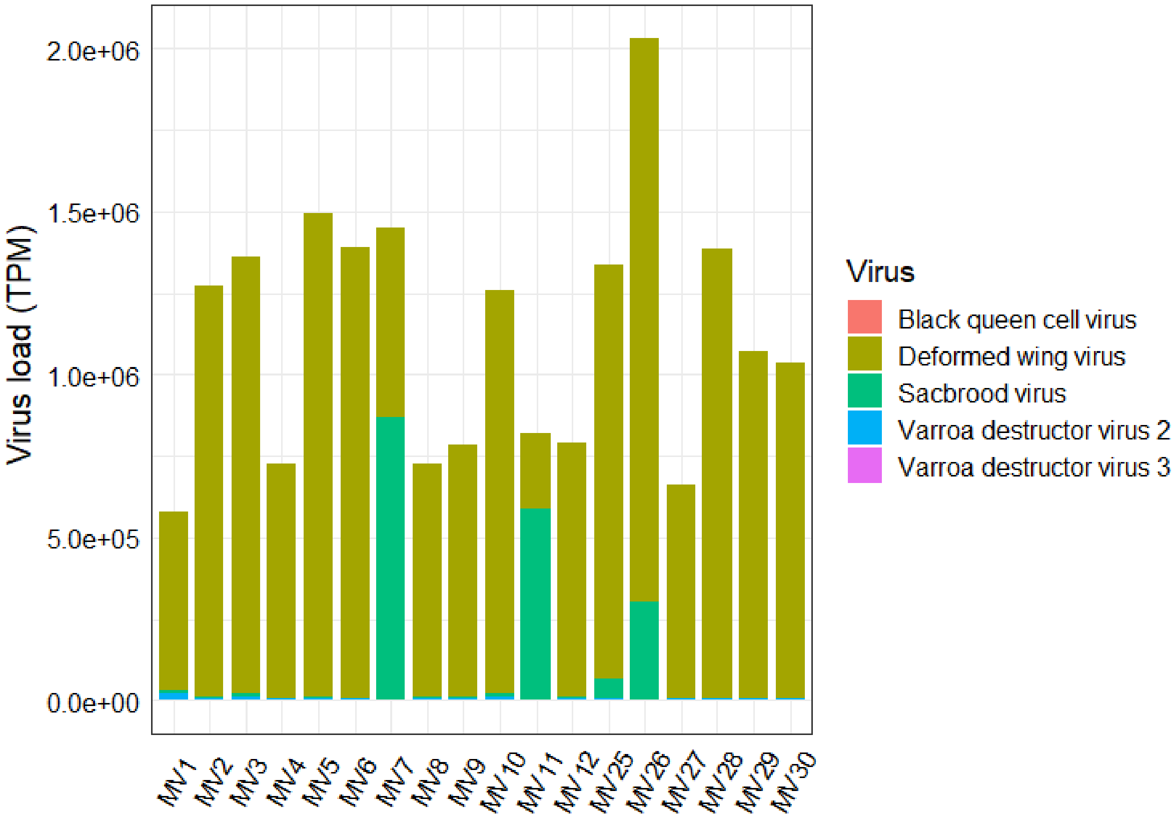

Figure S2. Relative abundance (transcripts per million; TPM) of viruses per RNA-seq varroa mite sample from each treatment; non-specific dsRNA (MV1-MV6), sugar control (MV7-MV12) and DWV-dsRNA (MV25-MV30).
