# Supplementary material for: Effects of Deformed Wing Virus-Targeting dsRNA on Viral Loads in Bees Parasitised and Non-Parasitised by Varroa destructor
Source: Viruses. 2023 Nov 15;15(11):2259. doi: 10.3390/v15112259 (PMC10674661; doi:10.3390/v15112259)
Supplement: Supplementary file 1 [file viruses-15-02259-s001.zip › FileS1.pdf]

Supplementary material: Effects of Deformed wing virus-targeting dsRNA on viral loads in bees parasitised and non-parasitised by *Varroa destructor*.

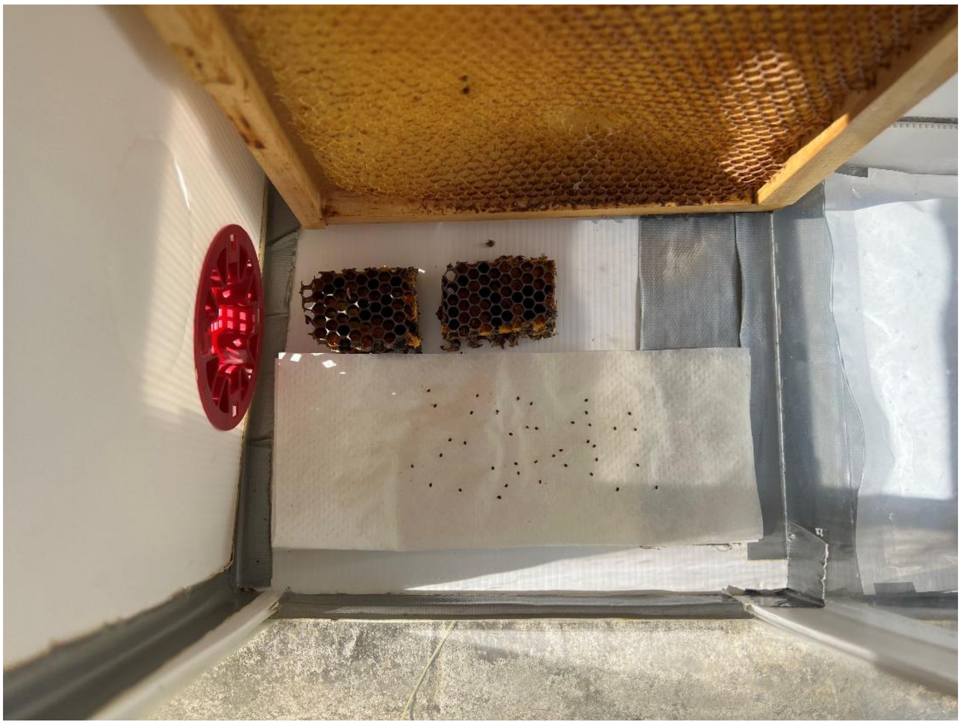

Figure S3. Brood chamber of mini-hive prepared to receive adult bees and a mini-frame of larvae. Mini-hives were prepared with an extra mini-frame of wax foundation, a paper towel containing introduced varroa mites and a square of stored bee bread.

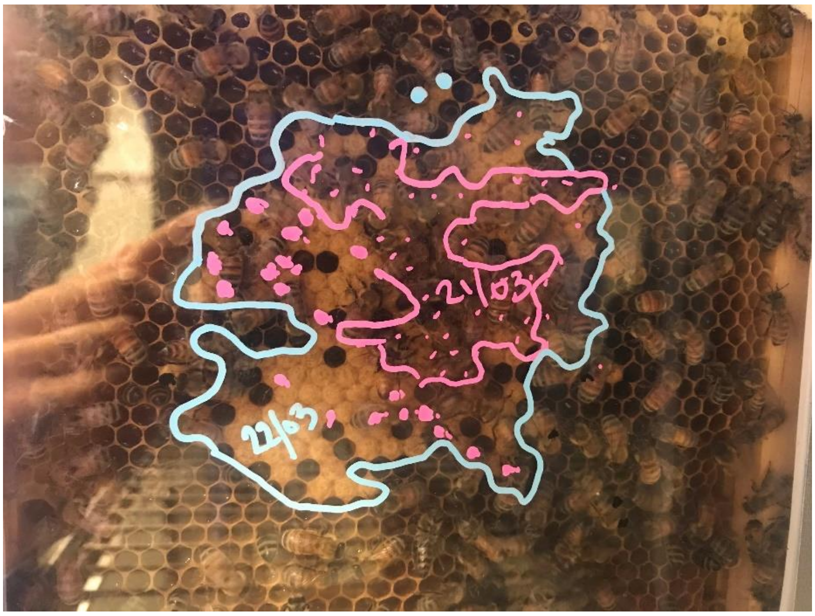

Figure S4. Capped cells were monitored closely in the lab. The plexiglass window was marked with the areas of capped cells and the date of cell capping. This allowed specific areas to be monitored for emerging bees. Frames were removed from the experiment and uncapped once bees were observed chewing the cell capping or at twelve days post capping.

Molecular methods

GeneZol™/ Chloroform-based RNA extraction method from individual bees

Tubes of bee homogenate were placed on a heat block at 65°C for 30 minutes, all liquid was then transferred to a new tube and heated at 65°C for 10 minutes. Tubes were then centrifuged at 16,000 rpm for 5 minutes. Supernatant (650 µl) was transferred to a new tube containing 650 µl of 24:1 chloroform/IAA mixture. Tubes were vortexed until a white emulsion appeared then centrifuged at 16,000 rpm for 5 minutes. Top layer of supernatant (500 µl) was carefully removed and added to a new tube containing 500 µl of cold isopropanol. Each tube was then inverted 10 times and either incubated at room temperature for 15 minutes or placed at 4°C overnight. Extractions were then centrifuged at 16,000 rpm for 20 minutes. Isopropanol supernatant was carefully poured off. The remaining RNA pellet was then exposed to two washes in 300 µl of 70% ethanol. Tubes were mixed enough to dislodge the pellet and centrifuged at 16,000 rpm for 5 minutes. As much of the ethanol supernatant was poured off as possible without losing the pellet between washes. Remaining ethanol was evaporated, and RNA pellets were resuspended in 150 µl of DNase/RNase free water. RNA concentration was then measured by NanoPhotometer NP80 (Implen, Germany).

Validation of quantitative PCR methods

Primer efficiencies were determined for both reference genes and DWVq by developing standard curves for each target using a 2-fold serial dilution of cDNA. All qPCR experiments were performed on an Applied Biosystems™ StepOne Real-Time PCR System. Each reaction consisted of 10 µl of PowerUp™ SYBR Green Master mix from Applied Biosystems™ and 3 µM of forward and reverse primers (Sigma-Aldrich). For each dilution in the 2-fold dilution series the following total amount of cDNA was added to the reaction: 20 ng, 10 ng, 5 ng, 2.5 ng, 1.5 ng, 0.625 ng per well. The following conditions were used to run the qPCR experiment: 2 minutes at 50°C, 2 minutes at 95°C followed by 40 cycles of 3 seconds at 95°C and 30 seconds at 60°C followed by a melt curve. For each target the mean cycle threshold (Cq) values of the duplicates for each dilution were plotted against the respective log10 dilution factor. A linear regression was conducted to determine the slope of the plotted values. Primer efficiencies for each target were calculated using the equation $E= (10^{[-1/slope]})$. Primer efficiencies for each target are shown in Table 5.

Table S5. Primers used in qPCR reactions for relative quantification of DWV loads in uncapped bees.

Primer	Target	Tm	Efficiency	Primer sequence	Reference
Ndufa8_Apis_F	Ndufa8	60°C	2.01	Forward: GCACGATTCACCAAGACCAA	Cameron, Duncan & Dearden 2013
Ndufa8_Apis_R				Reverse: GGTGGAGCTACAGGCTCAGG	
Pros54_Apis_F	Pros54	60°C	2.09	Forward: TCGAACCAAGATGGTACTGGAA	Cameron, Duncan & Dearden 2013
Pros54_Apis_R				Reverse: TTGTTGTGCTTGCAGTCGTG	
DWVQ_F	Deformed wing virus	60°C	2.14	Forward: AGTGCTGGTTTTCCTTTGTC	Martin et al. 2012
DWVQ_R				Reverse: CTGTGTCGTTGATAATTGAATCTC	
