# Supplementary material for: Effects of Deformed Wing Virus-Targeting dsRNA on Viral Loads in Bees Parasitised and Non-Parasitised by Varroa destructor
Source: Viruses. 2023 Nov 15;15(11):2259. doi: 10.3390/v15112259 (PMC10674661; doi:10.3390/v15112259)
Supplement: Supplementary file 1 [file viruses-15-02259-s001.zip › FileS2.pdf]

Table S6. Deformed wing virus double-stranded RNA sequences used in mini-hive experiment.

Double-stranded RNA	Region	Length (bp)	Sequence	Reference
DWV-dsRNA 1	RNA-dependent RNA polymerase	699	cacctggaacatcgggtaagcgatggttgtttgacattgagctacaagactcgggatgtt<br>atctcctgcgtggaatgcgtcccgaacttgagattcaattatcaacgacacagttaatga<br>ggaaaaagggaataaaacctcacactatattcacggattgtttgaaggatacttgtttgc<br>ctgttgaaaaatgtagaatacctggtaagactagaatatttagtataagtccggtacagt<br>ttaccataccgtttcgacagtattacttagactttatggcatcctatcgagctgcacgac<br>ttaatgctgagcatggtattggtattgatgttaacagcttagagtggacaaatttggcaa<br>caaggttgtctaagtatggcactcacatcgtgacaggagactataagaattttggtcctg<br>ggttagattccgatgttgcagcttcagcgttcgaaattattatcgactgggtattacatt<br>acaccgaagaagataataaagacgaaatgaagcgagtaatgtggaccatggcgcaagaga<br>tcttagcgcctagtcatctatatcgcgacttggtgtaccgagtaccttgcggaattccat<br>caggttctccaataacggacatattgaatacaatttcaaattgtttgttaattaggttag<br>cttggttaggtattactgatttgcctttgtccgagttct	Desai et al. 2012; Leonard et al. 2020
DWV-dsRNA 2	Helicase	443	gcatgagctagctttgcatccactctaccaatcggatataaatcggtatctaaaccaata<br>tcagattcatctaattcacgcaacggaagttcatacacacgatcatacggctctctttca<br>ctctcgattgctttaccggtgaacatttcatgtacaagtggttcagcaactccaaagcca<br>tgcaatccttcagtaccagcaacatggatacctataattggccgttgtaaattccgagac<br>ataacctcatacaaaccatcagcgttaatacttattggagttttattattattctcgaaa<br>gccaatagctgagtatggtcgccagttactaacactccatcattctgagcacgtatatgt<br>tcattatgtgacgctataaattt	Leonard et al. 2020
DWV-dsRNA 3	3C-protease	500	tattcatatacaatacgaataagcctttcccgaggtttgatcgtattgctatggaagcta<br>tttatcggcgtagaaatgttttgattgaatgtaaagcgagtgaagagaagaagcgaggat<br>gcaagcattgtgagaatgatattcctattgctgaatgtagtcctaagatgttgaaagatt<br>tccatcatatcaaatttaggtatgcacatgatgtatgtaattccgagaccacatggtctg<br>aatggatgacgtataatgaatttcttgaatggataactcctgtgtatatggctaaccgtc<br>gtaaggcgaatgaatcgtttaagatgcgtgtagatgaaatgcaaatgttacgtatggatg<br>aaccattggaaggtgataacattctcaataagtatgttgaagttaatcagcgcttagtgg<br>aggaaatgaaggcatttaaggagcgtacactatggtcagatttacatcgcttaggtgcgg<br>aaattagtgcgtcagttaag	Leonard et al. 2020

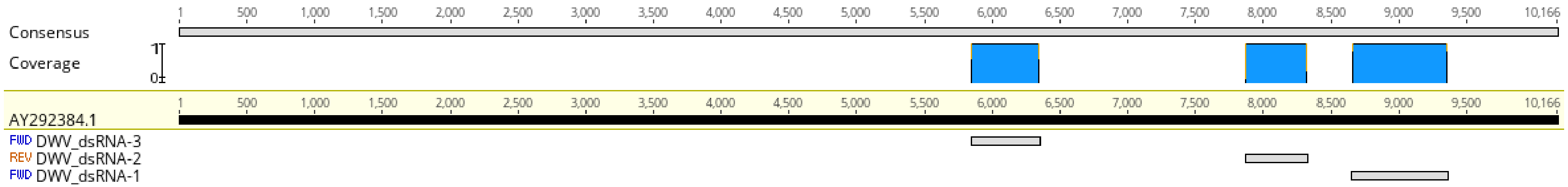

Figure S5. DWV-dsRNA sequences mapped to DWV-A reference genome (AY292384.1). used as a sequence template to design DWV-dsRNA sequences to show where in the genome the sequences target. Sequences were mapped in Geneious Prime 2020.2.3 (Biomatters, Auckland, New Zealand) where this figure was taken from.

References

Desai, S., et al., Reduction in Deformed wing virus infection in larval and adult honey bees (*Apis mellifera* L.) by double-stranded RNA ingestion. Insect Molecular Biology, 2012. 21(4): p. 446-455.

Leonard, S.P., et al., Engineered symbionts activate honey bee immunity and limit pathogens. Science, 2020. 367(6477): p. 573-576.
